# Unlocking Genome Editing: Advances and Obstacles in CRISPR/Cas Delivery Technologies

**DOI:** 10.3390/jfb15110324

**Published:** 2024-10-31

**Authors:** Bibifatima Kaupbayeva, Andrey Tsoy, Yuliya Safarova (Yantsen), Ainetta Nurmagambetova, Hironobu Murata, Krzysztof Matyjaszewski, Sholpan Askarova

**Affiliations:** 1 Center for Life Sciences, National Laboratory Astana, Nazarbayev University, Astana 010000, Kazakhstan; 2 School of Medicine, Nazarbayev University, Astana 020000, Kazakhstan; 3 Chemistry Department, Carnegie Mellon University, Pittsburgh, PA 15213, USA; 4 Department of Molecular Physics, Faculty of Chemistry, Lodz University of Technology, 90-924 Łódź, Poland

**Keywords:** CRISPR/Cas9, genome editing, functional biomaterials, synthetic polymers, lipid nanoparticles, cell-penetrating peptides, delivery strategies

## Abstract

CRISPR/Cas9 (clustered regularly interspaced short palindromic repeats associated with protein 9) was first identified as a component of the bacterial adaptive immune system and subsequently engineered into a genome-editing tool. The key breakthrough in this field came with the realization that CRISPR/Cas9 could be used in mammalian cells to enable transformative genetic editing. This technology has since become a vital tool for various genetic manipulations, including gene knockouts, knock-in point mutations, and gene regulation at both transcriptional and post-transcriptional levels. CRISPR/Cas9 holds great potential in human medicine, particularly for curing genetic disorders. However, despite significant innovation and advancement in genome editing, the technology still possesses critical limitations, such as off-target effects, immunogenicity issues, ethical considerations, regulatory hurdles, and the need for efficient delivery methods. To overcome these obstacles, efforts have focused on creating more accurate and reliable Cas9 nucleases and exploring innovative delivery methods. Recently, functional biomaterials and synthetic carriers have shown great potential as effective delivery vehicles for CRISPR/Cas9 components. In this review, we attempt to provide a comprehensive survey of the existing CRISPR-Cas9 delivery strategies, including viral delivery, biomaterials-based delivery, synthetic carriers, and physical delivery techniques. We underscore the urgent need for effective delivery systems to fully unlock the power of CRISPR/Cas9 technology and realize a seamless transition from benchtop research to clinical applications.

## 1. Introduction

**Figure 1:**
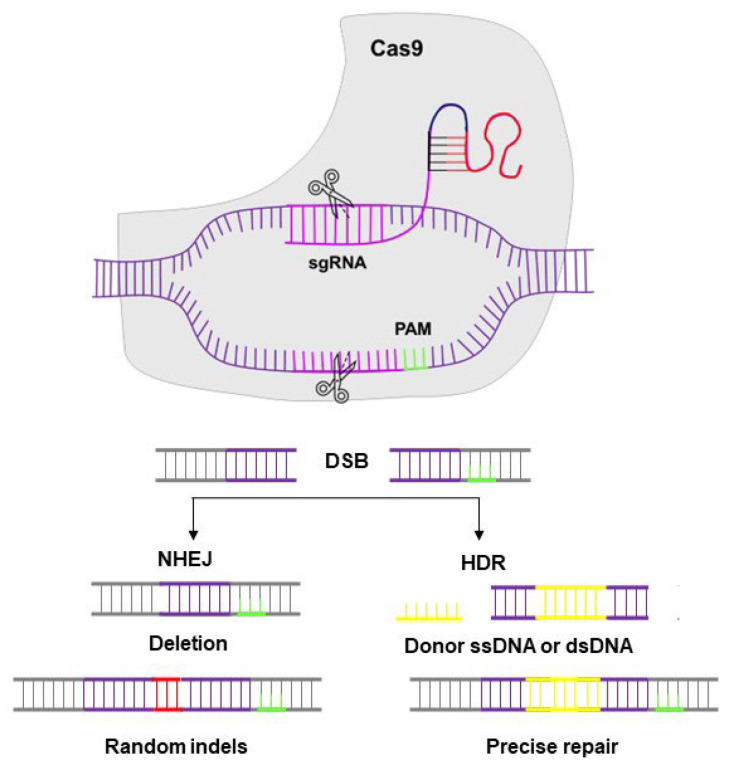
Schematic representation of CRISPR/Cas9-mediated gene editing. DSB—double-strand break, NHEJ—nonhomologous end joining, HDR—homology-directed repair, ssDNA—single-stranded DNA, dsDNA—double-stranded DNA.

Clustered regularly interspaced short palindromic repeats associated with protein 9 (CRISPR/Cas9) is a user-friendly gene editing platform. CRISPR/Cas9 was initially found as a prokaryotic defense system against mobile genetic elements such as plasmids or viral genetic material [1]. CRISPR/Cas9 is an essential trait enabling these microorganisms to evade the host’s innate immune system [2]. The CRISPR-mediated defense mechanism functions by inserting short DNA fragments of parasite genomes into the CRISPR array of the host to provide sequence-specific protection [3]. The Cas9 protein uses its single guide RNA (sgRNA) to selectively target any DNA site where the protospacer adjacent motif (PAM) is present [4,5,6]. Cas9 introduces site-specific double-stranded DNA breaks, which are then repaired by endogenous pathways in cells [7]. The sgRNAs can also be engineered to facilitate gene knockouts by delivering insertion or deletion mutations (indels) through nonhomologous end joining (NHEJ) or by promoting the integration of donor sequences via homology-directed repair (HDR) (Figure 1) [8].

Cas9 can be introduced into cells in the form of DNA, mRNA (transcribed from a plasmid), or purified protein, each with distinct advantages and disadvantages. Delivering Cas9 via plasmid DNA. which encodes both Cas9 and sgRNA, is a cost-efficient solution that requires only standard laboratory equipment for preparation. This can be advantageous if a longer-term need for Cas9 in the cell is required. However, the plasmid DNA must first be transcribed into RNA and then translated for Cas9 synthesis, which makes it more time-consuming than mRNA or protein methods. In addition, prolonged expression can increase the potential risks of off-target effects and insertional mutagenesis [9,10]. A faster mode of initiating gene editing is the delivery of Cas9 as mRNA, as it does not require transcription. Nevertheless, mRNA is unstable and quickly degraded by RNases. Thus, its expression is transient. Chemical modifications can improve mRNA stability, but transient expression might reduce gene editing efficiency [10,11]. Delivering Cas9 as a purified protein allows for immediate nuclear gene editing and, therefore, has higher efficiency than DNA- or mRNA-based methods. Unfortunately, protein delivery is the shortest-lived and most costly of all three formats, although it carries a lower risk of off-target effects. A fundamental challenge in Cas9 protein delivery is its bacterial origin, as it may carry bacterial endotoxins that trigger severe immune responses, limiting its clinical applications [12,13].

Several strategies for delivering Cas9 are currently being developed and tested. These strategies can be broadly categorized into four groups: viral vectors, biomaterials-based carriers, synthetic carriers, and physical methods.

Gene delivery using viral vectors is well established. While their wild-type versions cause diseases in humans, they have been engineered to be non-pathogenic while retaining the ability to robustly deliver genetic material into host cells without affecting their viability. The most utilized biological transfection vectors are adeno-associated virus (AAV), adenovirus (AdVs), and lentivirus (LV). Biomaterials-based carriers, also called nonviral vector-based delivery or chemical transfection for CRISPR/Cas9 systems, include cell-penetrating peptides, biopolymers, and lipid nanoparticles. In contrast, synthetic carriers are synthetic polymer-based delivery systems. Physical CRISPR delivery methods involve either applying direct physical mechanoporation (micro- and nano-injections, passing constrictions that can facilitate CRISPR delivery) or external forces such as electrical, acoustic, laser/thermal, or magnetic fields. These techniques permeabilize the cell membrane, enhancing the uptake of target molecules.

Each delivery method has its strengths and limitations, making their evaluation crucial when selecting the most appropriate approach for specific applications. Alongside successful delivery and high editing efficiency, safety is one of the most critical concerns in clinical trials. In this regard, our review introduces current CRISPR/Cas9 delivery strategies, with a focus on functional biomaterials and synthetic carriers, and discusses their applications in gene therapy, as well as their limitations and future prospects.

## 2. Viral Vectors for the Delivery of CRISPR Components

### 2.1. Adeno-Associated Virus Vectors

AAVs have been predominantly utilized to deliver the CRISPR/Cas9 system for several reasons. First, they have a good safety profile because their genomes remain episomal after the transduction [14]. Therefore, they have been approved for several human trials in gene augmentation therapies [15]. AAVs also display lower immunogenicity compared to other viral vehicles [16]. Additionally, even high doses of these molecules cause mild toxicity in animal models [17].

However, AAVs offer a limited packaging capacity of 4.7 kb, preventing the use of larger genes. Scientists enhanced the AAV delivery approach by developing a dual AAV system to address this issue. This expanded the capacity to approximately 9 kb [18]. Yang et al. illustrated the use of a dual AAV system to deliver the CRISPR/Cas9 technology, utilizing one vector to introduce SaCas9 and another to provide sgRNA targeting ornithine transcarbamylase (OTC) in mice with partial OTC deficiency, which affects the urea cycle in the liver [19]. Following the injection of two AAVs into newborn mice, the researchers found that the OTC mutation was reverted in 10% of hepatocytes, resulting in improved survival rates among the mice.

While using the dual AAV system addressed the limited carrying capacity found in a single AAV, specific genes still surpass the 9 kb limit imposed by this dual AAV strategy. Koo et al. demonstrated a trans-splicing method with triple AAVs to introduce the human dystrophin muscle dysregulation 1 gene (*DMD*) into dystrophic *mdx* mice [20]. Three separate AAVs were used to carry the *DMD* and the full-length gene was joined together upon inverted terminal repeats (ITR)-mediated trans-splicing. The results demonstrated the expression of human dystrophin in mice. Maddalena et al. showed retinal gene delivery through a triple AAV vector aimed at treating Usher syndrome type 1D and Alstrom syndrome, which are linked to mutations in the CDH23 and ALMS1 genes, respectively [18]. They demonstrated successful transduction of HEK293 cells, achieved 4% transduction of mouse photoreceptors, and 40% transduction of pig retina.

### 2.2. Adenovirus

Compared to AAVs, AdVs have a larger packaging capacity, reaching up to 8 kb [21]. Additionally, AdV is a non-integrating virus with an episomal genome, which minimizes undesired mutations caused by random insertions. Therefore, AdV-mediated delivery of CRISPR/Cas9 has been employed to establish disease models, generate resources for drug discovery, and address current health conditions.

For instance, in 2014, Maddalo et al. used intratracheal administration of AdV-delivered CRISPR/Cas9 to generate a model of non-small cell lung cancer by triggering the formation of an Eml4-Alk fusion gene [22]. Similarly, in 2015, Wang et al. created a mouse model of nonalcoholic steatohepatitis (NASH) by using AdV-delivered SpCas9 to target the Pten gene. The NASH phenotype emerged from the mutation in Pten and the immune reactions against SpCas9 [23]. In drug discovery, Voets et al. utilized AdV to disable the SMAD3 gene in normal human lung fibroblasts and bronchial epithelial cells using a low multiplicity of infection, as low as 20 [24]. In treating established diseases, Ding et al. demonstrated the reduction of plasma cholesterol levels by inducing loss-of-function mutations in the proprotein convertase subtilisin/kexin type 9 (PCSK9) gene in mouse livers [25]. There have been successful attempts to revive muscle function in muscle progenitor cells derived from patients with Duchenne muscular dystrophy in vitro [26] and in *mdx* mice in vivo [27,28]. Additionally, Li et al. engineered HIV-resistant primary CD4+ T cells by introducing the CCR5Δ32 variant into their cell membranes using AdV-delivered CRISPR/Cas9 [29].

However, AdVs pose a significant risk due to their high immunogenicity. Previous research has indicated that they provoke robust immune and inflammatory reactions in animal models, limiting their clinical potential [30]. Therefore, AdV vectors have been subjected to several optimizations. First-generation recombinant AdVs lack the viral gene E1 [14]. These vectors induce both acute and chronic immune responses. The viral capsid initiates the acute response, whereas the chronic response is triggered by viral gene expression. The second-generation vectors have been designed to reduce chronic immune responses by deleting viral genes E2 and E4. These vectors typically have a packaging capacity of approximately 8 kb [15,16]. The latest generation of AdV vectors, helper-dependent or gutless AdV vectors, lacks all viral genes except for inverted terminal repeats (ITRs) and encapsulation sequences. This design enables them to accommodate up to 35 kb of genetic material, making them well-suited for delivering the entire CRISPR/Cas9 system within a single vector [31,32]. Significantly, these vectors do not trigger long-lasting immune reactions. However, the viral capsid can still trigger acute phase immune responses. Additionally, since most individuals have been exposed to adenoviruses from early childhood and have developed neutralizing antibodies against common serotypes, the concentration of genes delivered in gene augmentation therapy typically decreases to about 10% within 24 months as a result of acute immune responses, and the presence of neutralizing antibodies [21,33]. This phenomenon ultimately limits the effectiveness of gene augmentation therapy [34].

### 2.3. Lentiviruses

LV vectors can accommodate genetic material up to approximately 7 kb in size, making them capable of hosting the SpCas9 gene, which is approximately 4.2 kb, along with additional sgRNA molecules. The production of LVs is also less complex than AAVs, and their transduction process is highly efficient across a broad spectrum of cell types [35]. In addition, LV vectors are devoid of all viral genes and do not trigger the immune response [36,37]. These advantages position LV vectors as an optimal choice for delivery in vitro and ex vivo compared to other viral vectors [35].

The lentiviral expression vector containing SpCas9 and sgRNA, utilized to alter targeted genomic sites, introduced a novel approach to assess gene function across the entire genome. For example, Shalem et al. demonstrated that delivering a genome-scale CRISPR/Cas9 knockout (GECKO) library targeting 18,080 genes using 64,751 sgRNAs allowed for positive and negative human cell selection [38]. These screens identified genes essential for cell viability in cancer and pluripotent stem cells. Another CRISPR/Cas9 library screening conducted with lentiviral vectors identified additional tumor-suppressing mutations through a functional search for targets associated with acute leukemia [39]. A new set of lentivirus vectors combined with CRISPR/Cas9 has been developed for lesion therapy in the treatment of HIV-1, HBV, and HSV-1 [40]. Furthermore, vectors have also been employed in correcting hereditary diseases such as cystic fibrosis and various neurodegenerative disorders [40].

However, the potential of LV systems for unpredictable incorporation into the host cell genome poses a challenge. Integration near oncogenes can activate them, potentially leading to tumorigenesis [41]. This limitation prevents LV-mediated delivery of CRISPR/Cas9 for in vivo gene editing in clinical studies [42]. Indeed, tragic incidents in clinical trials have been documented as a result of insertional mutations caused by retroviruses [43,44,45,46], underscoring the inherent risks associated with using LVs in patients.

Another common hurdle for using viral vectors is posed by the liver’s barrier function. Kupffer cells and liver sinusoidal endothelial cells have been shown to sequester most of the administered dose, thus preventing efficient delivery to the target tissues [47,48]. This results in the need for higher viral doses, which raises safety concerns. For instance, the use of high doses of AAV gene therapy led to the death of two pediatric patients [49]. To overcome this unintended liver capture of viral vectors, Kataoka et al. explored the direct stealth coating of liver sinusoidal wall cells with two-armed poly(ethylyene glycol)-conjugated oligo(l-lysine) (two-arm-PEG-Oligo-Lys) [50]. Preinjection of two-arm-PEG-OligoLys resulted in improved gene transfection efficiency in target tissues.

## 3. Biomaterials-Based Carriers

**Figure 2:**
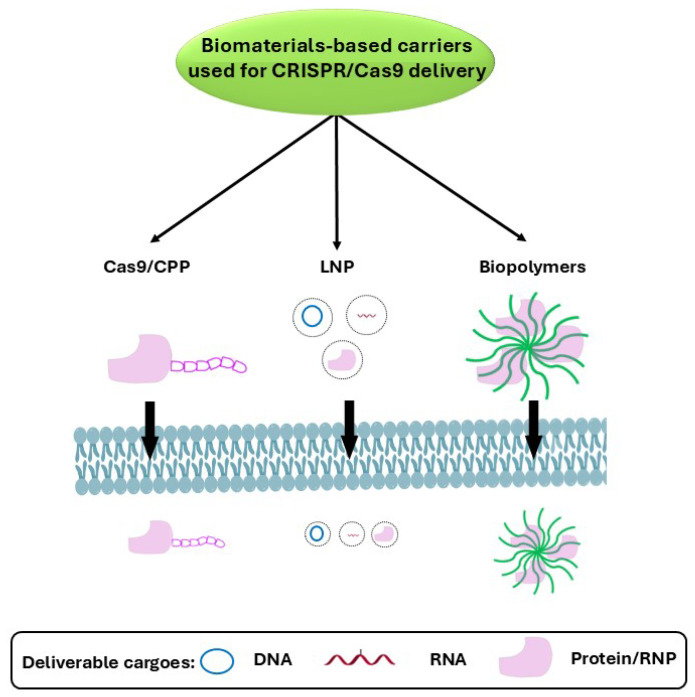
Biomaterials-based carriers used for the delivery of CRISPR/Cas9 systems.

Biomaterials-based carriers can be broadly categorized into three groups: cell-penetrating peptides (CPPs), lipid nanoparticles (LNPs), and biopolymers (Figure 2). These carriers have become increasingly popular for drug delivery due to their ability to control release, protect therapeutic payloads against degradation, potentially reduce immunogenicity/cellular uptake, etc., as well as add functionality to make active components bioavailable [47]. This has made biomaterials-based vectors a prevalent clinical platform for delivering low-molecular-weight drugs and protein/peptide therapeutics that have shown safety and efficacy in gene therapy through numerous clinical trials.

The first group of biomaterials used for CRISPR/Cas9 delivery, cell-penetrating peptides (CPPs), have shown great promise for improving the delivery of CRISPR/Cas9 components into target cells. These short, versatile peptides facilitate cellular uptake, allowing for efficient transport of the Cas9 protein and guide RNA. By leveraging the unique properties of CPPs, researchers can enhance the bioavailability and specificity of CRISPR/Cas9, potentially minimizing off-target effects and improving therapeutic effectiveness. The second class of biomaterials gaining considerable attention as CRISPR/Cas9 component carriers in cells are lipid nanoparticles (LNPs). These biomaterials encapsulate the Cas9 and gRNA, increasing their protection from degradation and enhancing cellular uptake. By utilizing LNPs, researchers can achieve targeted delivery and improved stability, potentially leading to more precise and efficient genome editing outcomes. The third group of biomaterials used for CRISPR/Cas9 delivery are biopolymers. These molecules rely on the non-covalent nanoparticle formation of polymers with CRISPR components. Biopolymer-based vectors offer several advantages, such as the ability to deliver multiple gRNAs at a time and the use of targeting ligands for functionalization with specific features that can facilitate CRISPR cargo delivery towards specific tissues and cells, including enough room for loading large payloads. Despite recent research showing some limitations in their ability to deliver CRISPR-Cas9 complexes, biopolymeric vectors may be inherently safe and have a large packaging capacity [51].

### 3.1. CRISPR/Cas9 Delivery by Cell-Penetrating Peptides

Cell-penetrating peptides (CPPs), also known as protein transduction domains, are short peptides capable of penetrating living cells. First discovered in 1988, the transcriptional protein HIV transactivator Tat was the first peptide sequence capable of crossing cellular membranes [52,53]. CPPs act as a delivery platform, carrying their cargo into the cell through perturbation of the cellular membrane lipid bilayer, or endocytosis [54,55]. Notably, CPPs such as HIV-1 Tat, penetratin, and oligoarginine are the best tools for transporting therapeutic biomolecules, generally termed biodrugs, into cells [56]. Researchers also investigated the capability of CPPs to act as penetration enhancers for poorly permeable polymers with low cell/membrane penetrability [57,58,59]. The precise processes of direct translocation, endosomal uptake, and endosomal escape of CPPs continue to be subjects of active investigation, with ongoing research into their applications in cargo transport [60,61,62,63,64,65,66,67].

A diverse array of CPPs with significant sequence variation exists, prompting extensive research into their chemical and biophysical properties that govern membrane permeation. These peptides are categorized based on their origin, function, sequence, uptake mechanism, and biomedical applications. Physicochemically, CPPs can be categorized into three main groups: amphiphilic CPPs, cationic CPPs, and hydrophobic CPPs [68]. Recent advancements in understanding CPP properties have been integrated into databases and software for predicting the cell entry capabilities of different CPPs [69,70,71,72]. General trends guiding these predictions include a preference for higher and lower arginine and lysine contents, as well as predictions of their secondary structures [70].

Over 40% of all CPPs identified are amphiphilic CPPs. Amphiphilic CPPs feature polar and nonpolar amino acid regions, with the nonpolar segment enriched in hydrophobic amino acids like alanine, valine, leucine, and isoleucine [73]. These peptides mainly facilitate intracellular transport and tend to concentrate in the nucleus [74]. Cationic CPPs possess a positive charge, allowing strong affinity with the cytoplasmic membrane under normal physiological pH conditions. Cationic CPPs bind to negatively charged cell membranes via electrostatic interactions and are transported into cells through a receptor-independent mechanism [75]. These CPPs typically possess a highly positive net charge, primarily composed of short basic chains of arginine and lysine, which play crucial roles in facilitating the internalization of various therapeutic cargoes [76]. The third category of CPPs consists of hydrophobic peptides characterized by a significant number of nonpolar amino acid residues. Hydrophobic CPPs are relatively uncommon and typically feature structures with a high proportion of nonpolar residues. These peptides possess hydrophobic amino acids, which are key in membrane translocation. Examples of hydrophobic CPPs are C105Y [77], Bip4 [78], Kaposi fibroblast growth factor (K-FGF) [79], and fibroblast growth factor 12 (FGF-12) [80]. Unlike many amphipathic cationic CPPs, the amino acid sequence of hydrophobic CPPs has been shown to have minimal impact on cellular uptake [78].

There are primarily two methods to conjugate CPPs with cargo. In the first method, the CPP/cargo sequence is expressed in *E. coli*, followed by the purification of the resulting recombinant fusion protein [81,82]. Ramakrishna et al. proposed incorporating a CPP into the SpCas9 protein [83]. However, genetically attaching SpCas9 with a CPP composed of four Gly, nine Arg, and four Leu made purifying the protein in adequate quantities challenging. To address this, a Cys residue was attached to the C-term of SpCas9 with minimal genetic modification. The primary amine (-NH_2_) group in the CPP attached through maleimide reacted with the free thiol group in the C-term cysteine of SpCas9, forming a CPP-SpCas9 conjugate through a thioether bond.

The second approach involves mixing CRISPR-RNPs with peptide reagents and applying them to cells in ex vivo culture [84,85]. Human cells, such as embryonic stem cells, dermal fibroblasts, HEK 293T cells, HeLa cells, and embryonic carcinoma cells, were exposed to the CPP-SpCas9 conjugate and CPP-sgRNA complex, either in a sequential or simultaneous manner. This CPP-mediated translocation system resulted in gene disruption ranging from 8.7% to 14% with minimized off-target effects, suggesting that CPP-mediated delivery could enhance CRISPR/Cas9 genome editing [84].

Using this method, genes were also knocked out in white blood cells by delivering Cas9 or Cas12a ribonucleoproteins or adenine base editors. Engineered cells demonstrated antitumor efficacy in mice, and the yield of edited primary human lymphocytes was significantly increased. This approach, named peptide-enabled RNP delivery for CRISPR engineering (PERC), enables highly efficient and precise multiplexed T-cell engineering, offering several benefits compared to other delivery technologies. PERC is effective with adenine base editors, Cas9, and Cas12a nucleases, indicating broad compatibility with various CRISPR effectors. It is as straightforward to introduce as viral transduction and allows for precise editing of multiple genes simultaneously. Furthermore, PERC has shown promise in correcting pathogenic mutations associated with inborn immune defects when used with base editing enzymes [85].

Although considered a cost-effective approach, CPPs show low editing efficiency, low specificity, limited stability, immunogenicity, and endosomal trapping. The methods require further optimization to advance the use of CPPs in clinical research. CPPs’ efficiencies to cross the cellular membranes vary depending on cell type and delivered cargo. Furthermore, challenges related to translocating the CRISPR/Cas9 complex into the nucleus must be addressed once it enters the cell.

### 3.2. Lipid-Based Delivery Systems

**Figure 3:**
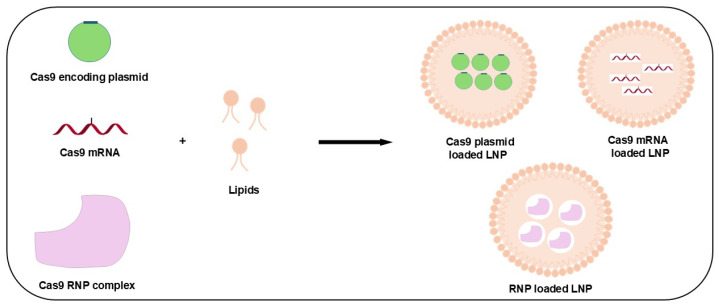
Schematic on the synthesis of LNP for CRISPR/Cas9 delivery system.

A promising strategy for drug delivery is to use advanced drug carriers known as lipid nanoparticles (LNPs), which encapsulate biological macromolecules, including nucleic acids (DNA or RNA) and proteins (Figure 3). Common approaches include enveloping the following: (1) plasmid DNA (pDNA) that encodes Cas9 and sgRNA, (2) Cas9 mRNA along with sgRNA, either together or individually, and (3) Cas9/sgRNA as a ribonucleoprotein (RNP) complex. Each method features unique benefits and drawbacks, necessitating specific formulation criteria for LNPs to maintain optimal compatibility and functionality [86].

Kulkarni et al. developed an LNP composition of pDNA encoding Cas9 and gRNA using DLin-MC3-DMA, replacing the saturated helper lipid distearoylphosphatidylcholine (DSPC) with unsaturated phosphatidylcholine (PC) helper lipids. They also assessed the effectiveness of various compositions containing ionizable positively charged lipids combined with unsaturated PC helper lipids. Among these, the most effective composition for primary mesenchymal embryonic cells derived from chicken embryos used DLin-KC2-DMA and 1-stearoyl-2-oleoyl-sn-glycero-3-phosphocholine (SOPC). This composition resulted in a transfection effectiveness of 90% and a cell viability of over 85%, in contrast to 50% transfection efficiency and 33% cell viability observed with lipofectamine [87].

Several challenges in pDNA delivery, such as volume of plasmid for encapsulation, penetration of cell membranes, nonspecific interactions with serum or extracellular proteins, and cytotoxicity, have been addressed by enhancing encapsulation efficiency. This was achieved by compressing the volume of plasmids using chondroitin sulfate and protamine to create a dense core [88]. Their LNP composition included cholesterol, the permanently cationic lipid 1,2-Dioleoyl-3-trimethylammonium propane (DOTAP), and the helper lipid 1,2-dioleoyl-sn-glycero-3-phosphoethanolamine (DOPE). Combining DOTAP and DOPE facilitated increased transfection by fostering electrostatic interactions with cellular membranes. Additional modification with DSPE-PEG decreased toxicity, enhanced stability and solubility, extended half-life, and minimized immune response. The ratio of DOTAP to DOPE was crucial in determining physicochemical parameters. This formulation was tested on cancer cells (A375 melanoma cell line, PC3 prostate cancer cell line, and MCF-7 breast cancer cell line) with overexpressed polo-like kinase 1 (PLK-1). Flow cytometry demonstrated approximately 47% transfection efficiency in A375 cells, and high-throughput sequencing identified 1 to 20 indels in the gene of interest. This composition demonstrated superior genome editing effectiveness in vitro and in vivo compared to lipofectamine [88].

Siegwart et al. have addressed the challenges of co-delivering different-sized Cas9 mRNA (4.5 kb) and sgRNA (0.1 kb) in vivo [89] by developing a selective organ targeting (SORT) strategy that enables tissue-specific gene editing. SORT can be engineered to target-deliver mRNA, Cas9 mRNA in complex with sgRNA, and Cas9 RNP to the lung, spleen, and liver in mice.

Targeting the PLK1 gene via LNP has successfully facilitated gene editing both in vitro and in vivo, even without plasmid condensation agents. Li et al. utilized a reporter gene system to transfect HepG2-Luc cells and mice with HepG2-Luc xenograft tumors using a novel ionizable LNP composition [90]. By adding a step to improve the stability of LNPs, this composition effectively encapsulated CRISPR/Cas9 pDNA targeting PLK1. This method resulted in a 32% reduction in mRNA levels in vitro, decreased tumor growth rate, decreased PLK1 mRNA expression levels, and increased programmed cell death in tumor cells in vivo [90].

There is an increasing emphasis on delivering Cas9 mRNA rather than pDNA for gene editing in both in vitro and in vivo applications. LNPs are being used to encapsulate Cas9 mRNA and gRNA together or separately. Finn et al. explored how modifying the tertiary structure of Cas9 sgRNA can improve editing effectiveness in vivo, explicitly targeting the Ttr gene, which is highly expressed in rare amyloidosis disorders [91]. In another study, Qiu et al. generated an LNP-mediated delivery system for Cas9 mRNA, targeting the liver to disrupt the angiopoietin-like 3 (Angptl3) gene [92]. The group employed bioreducible LNPs to deliver both Cas9 mRNA and sgRNA, demonstrating high in vitro editing efficiency and significant decrease of the Pcsk9 gene in vivo. The system featured a tail-branched bioreducible lipidoid (306-O12B) combined with excipient lipids to co-deliver SpCas9 mRNA and sgRNA targeting Angptl3 in a single administration. The 306-O12B LNP effectively delivered Cas9 mRNA and sgAngptl3 to hepatocytes in wild-type C57BL/6 mice, achieving an editing efficiency of 38.5% and a 65.2% reduction in ANGPTL3 protein levels in serum. Furthermore, Angptl3 knockdown in the liver resulted in substantial reductions in low-density lipoprotein cholesterol (LDL-C) and triglyceride (TG) levels, without any indication of off-target mutations or liver toxicity at the nine most likely predicted sites [92].

LNPs have been effectively used to deliver adenine base editor mRNA and sgRNA for targeting PCSK9 in vivo. An LNP composition similar to ONPATTRO, which encapsulates base editor mRNA and sgRNA targeting PCSK9, demonstrated up to 67% editing efficiency in rodents and s34% in macaques [93]. Notably, the editing was predominantly confined to hepatic tissues, with minimal off-target effects observed in non-hepatic tissues in both species, indicating the formulation’s tissue specificity.

Although there was a temporary rise in serum transaminase levels, indicating potential liver cell injury, the animals showed no symptoms, and the elevated levels resolved quickly [93].

Kenjo et al. employed an in vivo lipid screening technique to discover a new ionizable lipid, TCL053, for delivering mRNA via intramuscular injection [94]. Their LNP formulations with TCL053 encapsulated Cas9 mRNA and sgRNA separately. They were tested in a humanized mouse model of Duchenne muscular dystrophy (DMD), which involved a mouse model with Dmd1 exon 44 deletion and the addition of human DMD1 exon 45. This composition achieved approximately 10% exon skipping effectiveness in the tibialis anterior muscle, about five times higher than that observed with MC3-based LNPs. While serum cytokine levels increased 6 to 24 h after administration, these levels returned to normal within 7 days. The formulation’s low immunogenicity allowed for the safe readministration of the LNPs once, twice, or three times at monthly intervals, resulting in cumulative DMD1 editing [94].

Wei et al. created a technique for generating and engineering altered lipid nanoparticles that effectively deliver the Cas9/gRNA RNP complex to various tissues [95]. Intravenous administration facilitated tissue-specific, multiplexed modification of six genes in the lungs of mice. The high efficacy of these carriers was leveraged to develop cancer models specific to organs in the livers and lungs of mice through targeted gene deletions. Additionally, the RNPs successfully restored dystrophin expression in dystrophin-deficient mice and sharply reduced PCSK9 levels in the blood serum and livers of C57BL/6 mice with these advanced carriers [95]. The 5A2-DOT-10 LNPs, which encapsulated Cas9/sgDMD RNPs, were administered weekly into the Tibialis Anterior (TA) muscles of mice with a DMD exon 44 deletion, resulting in a 4.2% recovery of dystrophin protein as quantified by Western blot analysis. Furthermore, in the group treated with 5A2-DOT-5 LNPs that encapsulated Cas9/sgPCSK9 RNPs, the T7EI assay demonstrated successful indel formation at the PCSK9 gene loci [95].

Wang et al. modified their LNP composition to include bioreducible lipids that can effectively deliver polyanionic RNPs to green fluorescent protein (GFP)-expressing HEK cells [96]. These bioreducible lipids enhance the endosomal escape of the cargo, achieving more than 50% efficiency in reducing enhanced green fluorescent protein (eGFP) signals. Nuclear localization signals (NLSs) on Cas9 facilitate its efficient transport into the nucleus, followed by genome editing [96]. The study highlights that employing a combinatorial approach to screen distinct LNP formulations, including anionic proteins and the formation of Cas9 with polyanionic sgRNAs, could lead to developing a highly effective gene-editing delivery system [96].

Zuris et al. have developed a technique utilizing standard cationic lipid nucleic acid transfection reagents to effectively introduce polyanionic proteins into human cells, even in the presence of serum [97]. This method effectively introduced several proteins, such as Cre recombinase, TALE- and Cas9-based transcription activators, and Cas9:sgRNA nuclease complexes. Results demonstrated that unmodified Cas9:sgRNA complexes achieved up to 80% genome editing efficiency, demonstrating higher specificity than DNA transfection. Additionally, this technique has proven effective for delivering RNP proteins into the inner ear of mice in vivo, leading to significant recombination and genome editing within hair cells [97].

Although concerns regarding lower transfection efficacy, stability, and the potential to trigger immune responses remain [98], the rapid advancements in research on LNPs and their successful use in delivering mRNA in the Pfizer and Moderna vaccines for SARS-CoV-2 is expected to advance the clinical application of LNP carriers. Beyond the lipids used in COVID-19 vaccines, significant efforts are underway to generate safe and effective ionizable lipids to enhance in vivo delivery. Currently, Intellia Therapeutics is at the forefront of translating LNP-mediated delivery of CRISPR therapeutics using mRNA into clinical settings, and it is expected that the application of new LNP-RNP systems will broaden into various therapeutic domains in the future.

### 3.3. Biopolymers

Recently, biopolymers have been considered promising CRISPR/Cas9 delivery vehicles due to their biocompatibility and low toxicity [99,100]. Proteins (e.g., albumin, collagen), polysaccharides (e.g., chitosan, alginate), and nucleic acids are among the natural polymers being explored for this purpose [100]. Zhang et al. reported one of the first chitosan-mediated delivery systems for gene therapy [101]. Chitosan was pegylated with poly(ethylene glycol) monomethyl ether (mPEG). These copolymer conjugates demonstrated the ability to shield DNA from DNase I degradation and achieved maximum transfection effectiveness. PEGylated chitosan was capable of carrying DNA through the mucus model. This can be important in the realm of lung diseases.

In various studies, the chitosan derivative carboxymethyl chitosan is used due to improved solubility and protonation of its amino groups, making it suitable for pH-dependent release [102]. Furthermore, carboxyl groups could be modified with a ternary polymer to induce the targeting moiety of the gene delivery system [103]. Whereas in most systems, chitosan is not used as a single molecule for Cas9 delivery, the decoration of the polymer construct with chitosan helps endosomal transport through the cell membrane. For instance, in a study by Srivastav et al. [104], chitosan-coated PLGA nanoparticles showed profound efficiency in genome editing, providing 70% improvement compared to the control according to the experimental settings [105].

Alginate, a naturally derived polymer, is widely recognized for its stability and ability to form water hydrogels, making it appealing for biomedical applications. It is mucoadhesive and mucopenetrating, enhancing its effectiveness in drug delivery systems. Additionally, alginate is FDA-approved, further supporting its use in clinical settings. However, its limitations include low encapsulation efficiency and a short lifetime. Despite these challenges, alginate offers significant advantages, including a high affinity to the CD44 receptor and the ability to be easily functionalized. It is also degraded by hyaluronidase, which can be advantageous in controlled degradation and drug release scenarios. These properties collectively make alginate a versatile but sometimes limited choice for certain therapeutic applications. Alallam et al. employed alginate nanoparticles (ALG NPs) as a CRISPR/Cas9 gene editing delivery system. Using an electrospray technique, the researchers successfully encapsulated two CRISPR plasmids within ALG NPs, achieving a mean nanoparticle size of 228 nm and a zeta potential of −4.42 mV. The encapsulation efficiency exceeded 99% while preserving the integrity of the CRISPR plasmids. The CRISPR-loaded ALG NPs demonstrated cytocompatibility and successfully delivered the Cas9 transgene into HepG2 cells, effectively editing the GFP gene by inducing double-strand breaks [106].

Cyclodextrin is a versatile polymer known for its ease of modification, making it highly adaptable for various biomedical applications. It is particularly effective in encapsulating bioactive molecules, thereby improving the stability of these encapsulated cargoes. This enhances the efficacy and shelf life of the therapeutic agents it carries. However, cyclodextrin typically requires additional modification for applications involving DNA condensation, as its natural structure does not inherently support this function. Notably, a supramolecular polymer system using disulfide-bridged biguanidyl adamantine and β-cyclodextrin-conjugated poly(ethylene imine) effectively mediates the controlled intracellular delivery of Cas9 RNP, achieving high genome-editing activity in both 293T cells and colorectal cancer (CRC) cells [107]. Additionally, when decorated with hyaluronic acid (HA), this system successfully targets mutant KRAS, significantly suppressing tumor growth in mouse models and offering a prospective therapeutic strategy for colorectal cancer treatment [107]. Cationic polymers, such as polyethyleneimine-β-cyclodextrin, have efficiently delivered large plasmids that encode Cas9 and sgRNA, resulting in successful genome editing at specific loci [108]. These polymer-based systems provide advantages over viral vectors, including larger packaging capacity and improved safety profiles [51].

Protamine is another advantageous material for gene delivery studies and therapeutic applications owing to its high DNA condensation potential and inherent pharmacological activity. However, a significant disadvantage of protamine is its aggregating nature in circulation, which reduces efficacy and may cause adverse side effects. Despite this limitation, protamine remains essential in drug delivery due to its unique properties. A novel biomimetic core-shell system mimicking cell membranes was designed for light-controlled and precise gene editing. This system explicitly targets the elevated levels of hypoxia-inducible factor-1 alpha (HIF-1α) linked to cancer metastasis and resistance to treatment. This system combines protamine and calcium ions for efficient CRISPR/Cas9 delivery, with a hidden shell modified for tumor targeting and light-triggered gene editing. The system facilitates endosomal escape upon laser activation and releases the CRISPR/Cas9 assembly in the cytoplasm, allowing for precise gene editing. This approach significantly improves the therapeutic effectiveness of chemotherapy medications, reduces cancer spread, and offers an approach to cancer treatment. The system demonstrated enhanced antimetastatic effects in neoplastic H1299 cells and improved the in vivo efficacy of paclitaxel, offering a promising strategy for cancer therapy through precise gene disruption [109]. Another study highlighted an innovative approach to enhancing the delivery effectiveness of protamine-based transporters by incorporating lipids. A PEG phospholipid-altered positively charged lipid nanoparticle was developed, featuring a protamine, plasmid, and chondroitin sulfate core with a cationic lipid capsule. This design effectively shielded DNA and RNA from degradation. It facilitated release from endosomes, achieving a transfection efficiency of 47.4% in A375 cells in vitro and significantly suppressing tumor development in vivo through intertumoral injection [88].

While all the methods discussed above focus on utilizing polymer-based carriers to transport the RNP complex, an alternative and intriguing approach is directly modifying the Cas9 surface with polymers. In 2021, Kang et al. explored this by decorating the Cas9 surface with branched PEI, demonstrating that it could effectively complex with gRNA [21] and a DNA donor template for HDR [110]. The reported HDR efficiency was approximately 31%, with minimal cytotoxicity. This was the first Cas9 surface modification with polymers, which the authors called a “minimal carrier-assisted” delivery technique. This direct modification approach offers several advantages: polymers can continuously shield Cas9 from the immune system, enhancing biocompatibility. Additionally, polymers can stabilize the RNP complex and protect both the gRNA and donor DNA, potentially improving the effectiveness and safety of the gene-editing process.

Thus, polymers provide greater rigidity and stability than lipid nanoparticles (LNPs), and they can be engineered into various nanostructures with customizable sizes, charges, and compositions. The outer shell of polymer nanoparticles can be functionalized with unique ligands or antibodies, enabling precise delivery to particular cell types or tissues, which enhances the precision of CRISPR/Cas9 transport. Additionally, polymers are advantageous due to their low toxicity and minimal immunogenicity. Among polymers, natural biopolymers are often favored over synthetic ones for CRISPR/Cas9 delivery due to their superior biocompatibility and biodegradability, making them safer for in vivo applications.

## 4. Synthetic Polymer-Based Carriers

Recent research shows promising results in using synthetic polymer-based delivery systems for CRISPR/Cas9 gene editing. These benefits include customized design, reduced immunogenicity, direct functionalization, and high-yield fabrication without the difficulties of stoichiometric control between CRISPR components that are inherent to lipid-protein nanoparticles [111,112].

Polyethylenimine (PEI) is extensively utilized due to its strong cationic nature, making it ideal for gene transfection [113]. For instance, branched PEI-25K successfully delivered CRISPR/Cas9 plasmids to Neuro2a cells to target the Sck26a4 locus, mediating targeted gene editing [114]. PEI-functionalized magnetic nanoparticles effectively transfected HEK293 cells, achieving genome editing rates comparable to lipofectamine [115]. Polyethyleneimine-β-cyclodextrin (PC) efficiently delivered large Cas9/sgRNA plasmids, successfully editing two genomic loci: the hemoglobin subunit beta, achieving up to 19.1% editing efficiency, and rhomboid 5 homolog 1, with 7% efficiency [108]. Carbon dots conjugated with PEI1.8k and arginine (CD-PEI1.8k-Arg) outperformed native PEI in delivering CRISPR complex across various cell lines, maintaining efficiency in high serum concentrations and low plasmid doses. This system also effectively knocked out the GFP gene and showed potential for local brain tissue delivery [116]. Moradi et al. designed nanoparticles by combining an arginine-disulfide linker with low-molecular-weight polyethyleneimine (PEI1.8k), creating PEI1.8k-Arg nanoparticles. These nanoparticles enhance plasmid release and membrane permeability and facilitate nuclear distribution, resulting in improved transfection effectiveness compared to unmodified PEI1.8k.

The transporting system was influential not only in conventional cells (HEK 293T) but also in cells that are considered challenging to transfect, such as primary cells (HUVECs), cancer cells (HeLa), and neuronal cells (PC-12), achieving between 5- and 10-fold increases in transfection compared to the standard polymeric transfection agent [117]. These studies highlight the versatility and effectiveness of PEI-based carriers for CRISPR/Cas9 delivery, paving the way for improved gene editing applications. Li et al. proposed a method of precise genome editing that could be imaging-guided and stimuli-triggered, making the process visible and controllable [118]. The complex structure of the polymer construct with the alkyl side chains and fluorinated PEI formed the hydrophobic core that holds Cas9 via electrostatic interactions. Additionally, glucocorticoid dexamethasone was introduced into the polymer backbone to dilate the nuclear pores upon encountering the nuclear glucocorticoid receptors. The construct’s elegance is finely tuned through the photothermal transducing property of polymer brush chains that are triggered by laser irradiation. Studies conducted in vitro on HEK-293T cells and HCT 116 cell lines showed that the approach is not cytotoxic, and in vivo data confirmed that the strategy is viable, allowing for genome editing to be carried out under physically visible control [118].

Polylactic-co-glycolic acid (PLGA)-based nanoparticles can also potentially deliver CRISPR/Cas9 components for gene editing. Jo et al. proved that these nanoparticles carry large CRISPR-Cas9 plasmids and release them in primarily bone-derived macrophages within 24 h [119]. PLGA nanoparticles were exploited to efficiently deliver Cas9 protein and sgRNA to hematopoietic stem and progenitor cells, resulting in gene editing and enhanced fetal hemoglobin expression without causing cellular cytotoxicity [120].

Recent studies have used PEG-based transporting platforms for CRISPR/Cas9 gene editing. PEGylated nanoparticles based on α-helical polypeptides resulted in effective cellular uptake and release from endosomes, yielding a 47.3% gene editing efficiency in vitro and 35% knockout in vivo [121]. PEGylated PLANAs, a class of modified peptide/lipid-associated nucleic acids (PLANAs), exhibited reduced toxicity and a transfection efficacy equivalent to commercial reagents for CRISPR/Cas9 ribonucleoprotein delivery [122]. Liver-targeted polymeric micellar nanoparticles containing pCas9 (PEG-polycarbonate and cholesterol-modified PEI) exhibited superior cellular uptake, biocompatibility, in vivo retention within the tumor (via passive targeting), gene editing capability, and antitumor activity following IV administration to mice [123]. Moreover, Kataoka et al. demonstrated the challenging co-delivery of Cas9 mRNA and sgRNA using a poly(ethylene glycol)-shielded polyion complex by co-encapsulating sgRNA with Cas9 mRNA in a single platform [124]. Following intraparenchymal administration, this platform enabled efficient genome editing in mouse brain parenchymal cells.

Poly(beta-amino ester) (PBAE) polymers represent a novel type of nonviral vector for CRISPR/Cas9-based gene editing and other therapeutic nucleic acids. These biodegradable polymers have several advantages, including high transfection efficiency, large packaging capacity, and safety [125]. PBAEs can effectively deliver plasmid DNA encoding Cas9 and sgRNA for gene knockout and deletion [126] and base editors for precise gene editing [127]. Modifications to PBAEs, such as carboxylation and guanidylation, have enhanced their ability to deliver proteins and CRISPR/Cas9 ribonucleoproteins [128,129]. Recent advancements include cyclization-enhanced PBAEs for improved gene editing in disease models [130] and highly branched PBAEs for targeted genomic excision [51]. These developments emphasize the prospects of PBAEs as robust and efficient transporting vehicles for various gene editing applications in vitro and in vivo [131]. In a 2022 study by El-Kharrag et al., RNP complexes targeting the CD33 gene were transported to CD34+ cells utilizing polymer nanoparticles based on PBAEs, resulting in an 85% cell survival rate and a CD33 expression reduction ranging from 13% to 85%. Compared to electroporation, the nanoparticle delivery method showed higher cell survival (85% vs. 72%), more significant CD33 expression reduction (88% vs. 76%), and superior long-term multilineage engraftment potential [132].

Highly branched poly(β-amino ester) polymers have demonstrated potential in delivering CRISPR/Cas9 components, achieving up to 40% targeted genomic deletion in human cells [51]. Rui et al. reported a novel category of carboxylated branched poly(β-amino ester) capable of self-assembling into nanoparticles for effective cellular transport of CRISPR/Cas9 RNPs. In vitro, nanoparticles facilitated swift cellular uptake, effective endosomal release, and the delivery of functional proteins into the cytosol. RNP delivery in vivo triggered increased levels of gene editing at low RNP doses [128]. Authors suggest that encapsulating protein cargo within polymeric nanoparticles can reduce immune reactions by shielding the protein from circulating neutralizing antibodies. This protection may facilitate CRISPR gene editing in patients with an existing immune response. Overall, PBAEs possess key features for gene delivery, such as a reversible positive charge for nucleic acid binding, high buffering capacity, and rapid degradability. Still, their instability in physiological environments can limit transfection efficiency. However, their chemical versatility allows for modifications and blending with other materials to enhance stability and tailor them for specific delivery applications.

Poly [2-dimethylaminoethyl methacrylate] (pDMAEMA)-based polymers can efficiently condense plasmid DNA into nanoparticles, facilitating cellular uptake and transfection [133,134]. These polymers exhibit excellent transfection efficiency while maintaining low levels of cytotoxicity compared to commercial transfection reagents [135,136]. The proton sponge effect of pDMAEMA aids in endosomal release, contributing to its superior transfection capabilities [137]. Recent advancements include the development of hyperbranched and stimuli-responsive polymeric carriers for controlled CRISPR/Cas9 transport [136,138].

In response to the shortcomings of traditional polymer-based delivery systems, researchers have sought new strategies capitalizing on the virtues of both polymers and lipids. An promising alternative strategy involves synthesizing hybrid polymers, where lipids are grafted to a polymeric core-shell. These synthetic polymer-based delivery system advancements have demonstrated efficient transfection and gene editing capabilities, often surpassing commercial liposome-based reagents [135,138,139]. As a model system, the cationic lipid-assisted nanoparticle (CLAN) was optimized for macrophage delivery of Cas9 mRNA (mCas9) and sgRNA targeting NLRP3. This method resulted in the successful knockdown of NLRP3 gene expression that inhibited activation of the NLRP3 inflammasome upon diverse stimuli and uncovered the possibility of utilizing CLAN nano complexes as immune responses and modulatory tools through targeted gene delivery [140]. Zhang et al. used a similar strategy to enclose mCas9/gCD40 genes into PEG-b-PLGA-based CLANs for reprogramming dendritic cells to decrease transplant rejection [108]. Similarly, CLAN systems present inhibited netrin-1 expression in type 2 diabetic symptoms [141].

**Figure 4:**
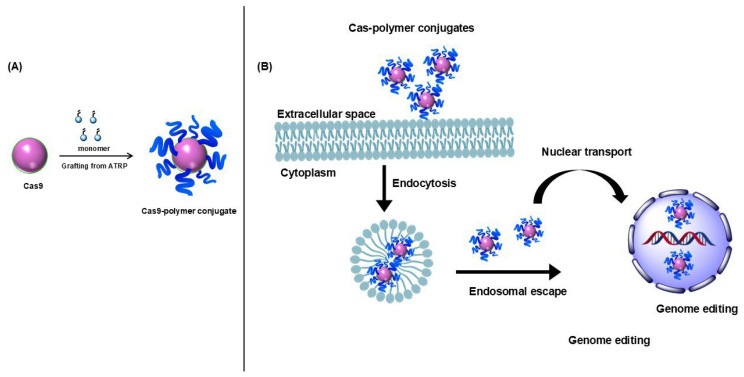
“Grafting from” Cas9. (**A**) ATRP-synthesis of Cas9-polymer conjugates. (**B**) Delivery of Cas9-polymer conjugates into cells.

While most polymer carriers encapsulate RNPs through electrostatic interactions, our current research focuses on covalent modification using a “grafting from” approach (Figure 4). This method employs atom-transfer radical polymerization (ATRP) to attach polymers to the surface of Cas9, modifying the enzyme with polymer chains. The advantage of this method is that it effectively immunoisolates and stabilizes both Cas9 and the sgRNA within the complex [142]. The “grafting from” technique allows for the growth of cell-permeable polymers to efficiently deliver the CRISPR/Cas9 system into cells [143]. Furthermore, our previous work has demonstrated that incorporating peptide linkers into the polymer sidechains, which can be cleaved by proteases, enables the controlled release of the enzyme core [144]. Similarly, Cas9 can be modified to facilitate its release in the nucleus. Additionally, incorporating nuclear localization signal peptides into the polymer sidechains can improve the complex’s ability to cross the nuclear envelope effectively. Overall, with the swift advancement in CRISPR technologies and the progress in nonviral delivery systems, these challenges are anticipated to be addressed shortly, paving the way for widespread integration of CRISPR/Cas9-based genome editing therapies into clinical practice.

The application of ATRP has emerged as a promising strategy in gene therapy [119,145]. Its robustness allows for precise control over polymer architecture [146,147], size [144], charge [148,149], and hydrophilicity [149], facilitating the synthesis of tailored carriers. Recently, ATRP has been developed to be more biocompatible by reducing the copper catalyst concentration required for the process and using low-energy green light irradiation [150].

Matyjaszewski et al. demonstrated that cationic nanogels containing DMAEMA and a cross-linker with reducible disulfide moieties, synthesized via ATRP, are highly effective for delivering pDNA and short interfering RNA (siRNA) [151]. Additionally, annealing complementary RNA strands with siRNA and conjugating with polymers from both sides can solve problems regarding endonuclease digestion and facilitate cellular uptake [152]. The researchers further showed the synthesis of block polymers with positively charged sulfonium (meth)acrylate and poly(ethylene oxide) using ATRP as a siRNA carrier. Block copolymers successfully formed polyplexes with siRNA and protected the siRNA against nuclease digestion. The efficiency of mRNA knockdown was evident from glyceraldehyde 3-phosphate dehydrogenase (*Gapdh*) decreased expression in murine preosteoblasts [153]. Hollinger et al. demonstrated that ATRP-synthesized cationic nanogel-siRNA polyplexes are efficient in in vivo knockdown of GFP in a mouse model [154].

ATRP also enables the synthesis of star polymers, which are widely used in gene delivery. These are branched polymers with multiple arms attached to one central core. Star polymers, consisting of poly(ethylene glycol) methyl ether methacrylate (PEGMA), peptide (Gly-Arg-Gly-Asp-Ser) modified PEG acrylate (GRGDS-PEG-Acryl), fluorescein *o*-methacrylate (FMA), and ethylene glycol dimethacrylate (EGDMA) were synthesized via ATRP [155]. These polymers demonstrated 90% cell viability after 24 h incubation with MC3T3-E1.4 cells. Additionally, peptide modification of the polymers resulted in 100% cellular uptake of the star polymers observed by flow cytometry. ATRP-prepared star polymers with PEGMA, DMAEMA, and a disulfide methacrylate cross-linker showed over 80% biocompatibility, even at high concentrations, and successful cellular uptake [156]. Further, star polymers synthesized from DMAEMA and EGDMA were combined with either pDNA or siRNA to form polyplexes [157]. This study demonstrated that low molar ratios of star polymers enabled the intracellular delivery of nucleic acids.

Recently, the synthesis of a new acylation agent that enables the incorporation of ATRP initiator into short synthetic RNA and natural biomass RNA was reported [158]. These RNA-initiator conjugates were subsequently used to grow polymers via photoinduced ATRP, allowing fine tuning of the properties of RNA-polymer hybrids. While creating nucleic acid-polymer conjugates via ATRP offers many applications, the synthesis is limited to grafting the polymers from the terminus of nucleic acids. To overcome this hurdle, Matyjaszewski et al. presented a novel serinol-based α-bromoisobutyryl (SBiB) phosphoramidite, which enables the multiple attachments of polymerization initiator groups at any site on an oligonucleotide [159]. This approach enables the synthesis a wider variety of nucleic acid-polymer hybrids. Moreover, the researchers demonstrated a way to circumvent the intrinsic instability of RNA molecules by adding protecting groups on the 2′-hydroxyl positions [160]. This resulted in the ATRP synthesis of highly stable RNA-polymer hybrids with the potential to expand the field of RNA-based polymeric materials.

Das et al. developed a method of modifying the surface of exosomes with oligonucleotide tethers [161]. They showed that the cellular uptake of the exosomes could be adjusted with tethered aptamer while the targeting specificity could be adjusted with tethered protein. Notably, the developed strategy is independent of the exosome source, making it universally applicable.

The development of a fluorescent nanotag capable of detecting protein targets in confocal microscopy and flow cytometry has been demonstrated by ATRP synthesis of bottlebrush polymers with hundreds of duplex DNA strands [162]. Hundreds of fluorescent dyes were covalently attached to DNA duplexes and tethered to a single antibody probe without risking self-quenching.

Matyjaszewski and Das demonstrated the synthesis of protein-polymer conjugates with noncovalent bonds mediated by DNA [163]. Despite being noncovalent, the DNA-mediated method offers precise pairing and assembly characteristics inherent to DNA. The same group reported the solid-phase synthesis of DNA-polymer hybrids, broadening the scope of advanced polymer-bioconjugate functional materials [164].

In summary, ATRP has proven to be a robust and versatile method for synthesizing polymers with precise control over their properties, making it a valuable tool in gene therapy. Its ability to create customized carriers and facilitate efficient nucleic acid delivery underscores its significant potential in advancing therapeutic applications.

## 5. Physical Transfection Methods for CRISPR/Cas9 System Delivery

### 5.1. Mechanoporation

Mechanoporation uses mechanical or shear forces to generate pores in cellular membranes. Microinjection is a conventional method of mechanical transfection for delivering CRISPR/Cas9 into cells [165,166,167]. This method injects CRISPR/Cas9 directly into cells using a microneedle under microscopic visualization. Direct transfer via microinjection of the CRISPR/Cas9 complex provides the benefit of transporting the entire CRISPR/Cas9 system, including components of various sizes, such as proteins. This method bypasses the limitations associated with size constraints that may exist with other delivery approaches. For instance, Ma et al. [168] demonstrated that microinjection was effectively used to knock out four genes (ApoE, B2m, Prf1, and Prkdc) in rats in a single step. The microinjection technique achieved this by co-injecting Cas9 mRNA and sgRNAs into zygotes. In a similar study, sgRNA has been effectively introduced alongside Cas9 protein targeting ovine myostatin (MSTN) by microinjecting it into the cytoplasm of ovine zygotes, aiming to disrupt the MSTN genomic sequence across the entire embryo; this approach effectively knocked out MSTN in sheep [166]. However, despite its effectiveness, microinjection presents challenges due to its demanding technical requirements, as it involves manually injecting a single cell [169].

One way to overcome the limitation of single-cell transfection is a microfluidic-based biophysical mechanoporation technique that involves passing several cells at increased velocities through narrow micrometer-scale openings. This process disrupts the plasma membrane, delivering different cargoes directly across the cytosol of various cells. For example, Sharei et al. utilized microfabricated silicon channels to generate temporary openings for introducing materials into cells [104]. Their method involved passing a mixture of delivery materials and cells through a microfluidic channel containing a constriction with a width of approximately 30–80% of the diameter of the cells. This approach facilitated the formation of temporary pores in the cells, enabling efficient delivery of materials. In a similar study, Han et al. developed a series of microfluidic silicon devices featuring a series of narrowing channels with varying dimensions formed by structures of different shapes [170]. The study showed that this method effectively facilitated the delivery of CRISPR components into traditionally challenging cell types like immune and stem cells.

Navigating cells towards nanoscale cell-penetrating structures is another mechanoporation technique that efficiently transfects larger numbers of cells by directly penetrating the cell membrane. In particular, silicon nanoneedles measuring 200 nm in diameter and coated with Cas9-sgRNA have been employed successfully [171]. Furthermore, silicon nanoneedles have been employed to convey plasmid DNA-encoding vascular endothelial growth factor into mouse muscles, thereby enhancing tissue neovascularization [172]. In this study, cells were interfaced with nanoneedles either by seeding them over the nanoneedles or pressing them over a monolayer of cells [172]. These nanoneedles puncture the plasma membrane, enabling the delivery of Cas9 complexes into the cells for targeted gene editing or manipulation. A key advantage of this strategy is that it transfects adherent cells in a monolayer rather than requiring them to be in suspension.

However, all the abovementioned mechanoporation techniques typically introduce materials into the cytoplasm, necessitating following transport of these materials into the nucleus for effective genome editing, which can lead to undesired transcription reactions. While specific research groups have demonstrated the ability to use the microinjection technique to directly deliver CRISPR/Cas9 constructs into the nucleus [173], this approach is low throughput and requires a highly skilled technician. A method called force-controlled fluidic injection (FCFI) into single-cell nuclei for improved CRISPR delivery has been developed to tackle this challenge. FCFI technology combines microfluidics and force microscopy by incorporating microscopic channels into force-sensitive probes. This integration allows for precise handling of liquid volumes at the femtoliter scale and enables force-controlled manipulations of microscopic objects, offering improved control over CRISPR delivery into cell nuclei [174]. However, micro- and nano-injections restrict their use for in vivo transport due to the efficiency of transfection being limited to individual cells.

### 5.2. Electroporation

Electroporation is the second extensively utilized physical transfection method for delivering genetic materials into cells [86,175]. This technique involves the application of a strong electric field across a cell membrane. The electric field surpasses the membrane’s capacitance, leading to the transient opening of nanometer-sized pores or swift disruption of the lipid bilayers, thereby facilitating efficient intracellular transport. Since this method does not require microinjection skills, it allows for the simultaneous treatment of a considerably greater amount of cells or embryos. For instance, Hashimoto et al. achieved effective genome editing in mouse embryos by optimizing the electroporation conditions for introducing RNAs of the CRISPR/Cas9 complex into zygotes [176].

Similarly, Alghadban et al. noted enhancements in mutagenesis efficiency for producing ssODN repair templates, along with increased rates of embryo survival and development, when administering CRISPR/Cas9 systems as ribonucleoproteins (RNPs) through zygote electroporation [177]. Wu et al. corrected a cataract-inducing mutation by CRISPR-Cas9-mediated gene modification in stem cells of spermatogonial mouse [178]. Rathbone et al. studied the impacts of electroporating Cas9 RNPs targeting Hpd, the gene encoding hydroxyphenylpyruvate dioxygenase, into primary mouse and human hepatocytes. They reported increased levels of gene editing in both cell types [179].

However, electroporation technology demands specific conditions tailored to different cell types. Applying strong currents can damage cells through excessive heat exposure, ionic imbalances, and pH alterations, rendering this method unfit for cells sensitive to stress. Therefore, meticulous evaluation and identification of suitable conditions are imperative for electroporation to achieve effective transfection while preserving cell integrity.

Xu et al. devised a novel tube-shaped cuvette to address several drawbacks associated with electroporation. This method targeted the knockout of β2-microglobulin (B2M), a crucial component of major histocompatibility complex (MHC) class I molecules, within primary human mesenchymal stem cells. The study demonstrated a remarkable 80.2% reduction in B2M expression by delivering Cas9/gRNA RNP and a single-stranded oligodeoxynucleotide (ssODN), introducing a frameshift mutation. Additionally, the technique significantly decreased the surface expression of the protein in mesenchymal stem cells, dropping from 95.6% to 59.9% [180]. In a separate investigation, nanostructure-mediated electroporation has facilitated the downsizing of physical transfection methods, leading to improved efficiency and precision. Moreover, it ensures uniform treatment of cells with minimal effects on viability, contrasting with the uneven cell permeabilization associated with bulk electroporation [181]. Notably, in gene editing applications in vivo, electroporation has effectively transported genetic materials into the mouse retina [182].

It is worth noting that all of the abovementioned studies were conducted using in vitro and ex vivo approaches. There are only a few reports of successful application of electroporation techniques for delivering Cas9-mediated systems in vivo. For example, Wu et al. utilized electroporation on mouse tail skin to restore C7 function in recessive dystrophic epidermolysis bullosa (RDEB) mice. They noted a rise in epidermal–dermal adhesion from 30% to 60% within 3 to 5 days following a single administration. Nevertheless, FACS analysis of tdTomato+ epidermal cells treated using this strategy showed fluorescence in 2% of the cells. Moreover, the enhanced epidermal–dermal adhesion observed in treated mice was not maintained beyond 5 days, raising concerns about the longevity of these gene edits [183].

While the success of the electroporation technique and its modifications hold promise for CRISPR/Cas9 gene editing for in vivo applications, several significant limitations need to be addressed before it can be considered safe and efficient for human use in clinical settings. These limitations include potential damage to surrounding tissues associated with the procedure and a limited comprehension of off-target effects. To overcome the limitations associated with high-voltage electroporation, researchers are exploring alternative methods of cellular poration, such as thermoporation [184], optoporation [185], and acoustoporation [186], which aim to retain the scalability and ease of use of electroporation while mitigating its drawbacks.

### 5.3. Hydrodynamic Injection

While mechanoporation and electroporation are primarily used with in vitro or ex vivo cultured cells, hydrodynamic injection has been successfully employed for in vivo CRISPR/Cas9 complex delivery. Hydrodynamic injection entails administering a substantial volume of solution containing the CRISPR/Cas9 system into the animal’s circulatory system [187]. This sudden rise in hydrodynamic pressure temporarily enhances the permeability of endothelial and parenchymal cells, enabling the CRISPR/Cas9 complex to penetrate cells in various tissues, such as the heart [85,86], liver [87,88,89,90,91], and lungs [188]. As an example, Schuh et al. demonstrated that hydrodynamic injection of a liposomal complex transporting the CRISPR/Cas9 plasmid and a donor vector in newborn Mucopolysaccharidosis type I mice resulted in a remarkable rise in serum alpha-L-iduronidase levels (IDUA) for up to 6 months. After hydrodynamic injection, they observed a marked biodistribution of the complexes in the lungs and heart. This supported the findings of elevated IDUA activity and decreased accumulation of glycosaminoglycans, particularly in these tissues [188]. However, this technically simple method can be rather distressing to the organism, often causing cardiac impairment, liver enlargement, and increased blood pressure [189]. To mitigate the hemodynamic side effects due to the rapid administration of a large volume, it is vital to moderate the conditions to ensure safety. One effective strategy is to bypass the blood vessels of the target organ and directly inject the solution. This approach achieved through catheterization and surgical techniques in large animals has allowed protein expression levels in tissues to resemble closely those achieved in gold-standard models. These potential outcomes in delivering CRISPR constructs have sparked renewed clinical enthusiasm in the gene transfer technique [189].

In summary, while physical transfection methods offer advantages, they have significant limitations for in vivo applications due to their invasive nature, making them primarily suitable for in vitro systems. Nevertheless, recent advancements in microtechnology and nanotechnology have introduced innovative physical transfection methods, presenting potential opportunities to expand the application of these techniques for use in human settings [190,191].

## 6. Conclusions and Future Perspectives

A significant milestone in CRISPR/Cas9 technology was achieved in 2023, when the US FDA approved the first CRISPR/Cas9-edited cell-based gene therapy, Gasgevy™ (Vertex Pharmaceuticals), for treating sickle cell disease and beta-thalassemia in patients aged 12 years and older [192]. Despite the remarkable achievements in gene therapy through the CRISPR/Cas9 system, ensuring the safe and effective application of this technology presents a conundrum. The most pressing challenge of this technology remains its delivery systems. Although several different techniques are currently being employed to deliver CRISPR into cells, each comes with its own strengths and weaknesses (Table 1). Therefore, the need for a universal platform for CRISPR/Cas9 has become more urgent than ever.

Generally, the most efficient gene editing outcomes with limited off-target effects are achieved by providing the RNP complex rather than plasmid DNA or mRNA. However, the direct delivery of RNP complexes presents several challenges. First, the hydrophobicity of the nucleic acids means they do not readily dissolve in water and cannot pass through cell membranes efficiently. Second, the negative charge that the phosphate backbone gives nucleic acids can limit their ability to be delivered into cells, as the cell membrane is also negatively charged. Additionally, nucleic acids are easily degraded by enzymes, mainly nucleases from plasma and intracellular nucleases, which limits their in vivo stability and efficacy in reaching target cells [193]. The immune system may further complicate delivery, as nucleic acids and bacterial-origin enzymes are potentially immunogenic, producing antibodies against both the nucleic acids and Cas9. To overcome these challenges, delivering CRISPR/Cas9 RNP complexes via functional biomaterials and synthetic carriers may significantly enhance the efficiency and potency of gene editing applications.

## Figures and Tables

**Table 1:** Characteristics of CRISPR/Cas9 delivery systems.

Delivery Method	Delivery Formats	Advantages	Disadvantages	Application
Adeno-associated virus vectors (AAV)	DNA	Low immunogenicity even at high doses	Limited packaging capacity requires more than one vector	Gene therapy for various genetic diseases
Adenoviral vectors (AdV)	DNA	Increased packaging capacity within one vector	Triggers acute immune response	Gene therapy for genetic diseases
Lentiviral vectors (LV)	DNA	Increased packaging capacity and low immunogenicity	Undesirable off-target effects observed in conjunction with CRISPR-Cas9	Primarily utilized for the development of screening tools
Lipid nanoparticles (LNPs)	DNA, mRNA, RNP	Low off-target effects, easy to produce, low cost, FDA-approved	Endosomal degradation of cargo, requires optimization	In vitro and in vivo genome editing
Cell-penetrating peptides (CPPs)	RNP	Low off-target effects	Low efficiency, immunogenicity	Genome editing for cells in vitro
Polymer-based delivery	DNA, mRNA, RNP	Low off-target effects, easy to produce, large packaging capacity	Variable efficiency, cytotoxicity	In vitro genome editing

## Data Availability

Not applicable.
